# Three-Dimensional Force Measurements During Rapid Palatal Expansion in *Sus scrofa*

**DOI:** 10.3390/mi7040064

**Published:** 2016-04-12

**Authors:** Kelly Goeckner, Venkatram Pepakayala, Jeanne Nervina, Yogesh Gianchandani, Sunil Kapila

**Affiliations:** 1 Department of Orthodontics and Pediatric Dentistry, School of Dentistry, University of Michigan, Ann Arbor, MI 48109, USA; goeckner@umich.edu (K.G.); jnervina@me.com (J.N.); skapila@umich.edu (S.K.); 2 Center for Wireless Integrated MicroSensing and Systems, Department of Electrical Engineering and Computer Science, University of Michigan, Ann Arbor, MI 48109, USA; yogesh@umich.edu

**Keywords:** dental, force measurement, maxillary expansion, strain sensing

## Abstract

Rapid palatal expansion is an orthodontic procedure widely used to correct the maxillary arch. However, its outcome is significantly influenced by factors that show a high degree of variability amongst patients. The traditional treatment methodology is based on an intuitive and heuristic treatment approach because the forces applied in the three dimensions are indeterminate. To enable optimal and individualized treatment, it is essential to measure the three-dimensional (3D) forces and displacements created by the expander. This paper proposes a method for performing these 3D measurements using a single embedded strain sensor, combining experimental measurements of strain in the palatal expander with 3D finite element analysis (FEA). The method is demonstrated using the maxillary jaw from a freshly euthanized pig (*Sus scrofa*) and a hyrax-design rapid palatal expander (RPE) appliance with integrated strain gage. The strain gage measurements are recorded using a computer interface, following which the expansion forces and extent of expansion are estimated by FEA. A total activation of 2.0 mm results in peak total force of about 100 N—almost entirely along the direction of expansion. The results also indicate that more than 85% of the input activation is immediately transferred to the palate and/or teeth. These studies demonstrate a method for assessing and individualizing expansion magnitudes and forces during orthopedic expansion of the maxilla. This provides the basis for further development of smart orthodontic appliances that provide real-time readouts of forces and movements, which will allow personalized, optimal treatment.

## 1. Introduction

Rapid maxillary expansion is a common procedure in orthodontics used to increase maxillary arch length and width by separating the maxillary bones along the midpalatal suture [1]. The opening of the midpalatal suture is also accompanied by distraction of bones at other facial sutures and changes at the cranial base [2,3]. Generally, rapid maxillary expansion is most successful in growing children who have patent or unfused sutures. While several different designs of tooth- and bone-borne expanders are available, the most commonly used appliance is the hyrax rapid palatal expander (RPE) that is typically attached to two teeth on each side of the maxillary arch. 

Although RPEs are largely thought to deliver force vectors in two rather than three dimensions—a postulate that remains to be examined—they function within a complex three-dimensional (3D) craniofacial structure composed of a number of bones that are separated by sutures, that in turn reside within an environment that includes active muscles and soft tissues [4]. Furthermore, the RPE is attached to teeth that undergo dental movements due to the forces that also result in the modeling of bone. Together these lead to shortcomings in treatment with RPE that include dental movements as opposed to preferred skeletal movements, dental tipping, lack of optimal expansion in different areas of the palate, and the development of boney fenestrations and post-treatment relapse [2,5,6,7,8,9]. This complex and dynamic environment contributes to a lack of a detailed understanding of how tissues respond to RPE expansion forces and compromises the predictability of the planned treatment outcomes.

Given these complexities, many variables must be taken into account in planning treatment with an RPE. These include, but are not limited to, sutural maturation, soft tissue and/or scar tissue resistance, the magnitude and types of movement of teeth to which the RPE is anchored, the location of the RPE in all three dimensions relative to the maxilla and teeth, and expected relapse after expansion [4,10,11,12]. All of these variables would determine the magnitude and direction of forces required to obtain predictable, efficient, and stable expansion of the maxilla. Unfortunately, little is known about the precise nature of forces needed to optimally expand the palate during RPE treatment. Instead, treatment is typically rendered on the basis of the desired magnitude of expansion for each patient rather than the expected responses to the expander and regardless of the forces experienced by the maxillary structures.

In addition to measuring the total expansion force, it is critical to understand the forces generated in all three spatial dimensions (*x*, *y* and *z*). Isaacson *et al.*, measured maxillary expansion force in humans and concluded that there is high variability in both the expansion force and its temporal evolution [13,14,15]. However, this investigation only reported total expansion force and did not resolve the force in three dimensions. Knowing the expansion forces exerted in three dimensions with a conventional RPE will allow for custom designs and individualized treatment planning to generate optimal forces in the desired directions. Optimizing the expansion force requires knowledge of the magnitude and direction of expansion generated by the RPE specifically for each patient. This can be achieved by accurately measuring the expansion force generated by the activation of the RPE using an integrated sensor to provide the orthodontist access to real-time data for each patient, thus facilitating customized force levels and treatment modalities. Such real-time information would enable the clinician to determine whether optimal forces for sutural expansion have been achieved, the magnitude and rate of force decay following activation, and optimal timing and amount of reactivation of the appliance specific to each individual.

Specific animal models have been utilized to study and refine protocols and technology for maxillary expansion prior to utilizing these approaches on humans. *Sus scrofa* and Yucatan minipigs are often chosen as experimental models for RPE studies because a pig’s midpalatal suture anatomy and chewing patterns, and the resulting transmission of force, are similar to humans [16]. Porcine bone is also physiologically and metabolically similar to human bone in terms of bone mineral density, bone mineral concentration and bone remodeling [17]. When using pigs for RPE studies the pig’s age is important because enough tooth structure must be fully erupted into the maxillary arch to stably affix the tooth-borne RPE, while still having an appropriately immature maturational status to ensure that a patent midpalatal suture that will respond favorably to expansion forces.

Although a tooth-borne RPE is expected to have the applied force manifest in three dimensions that would be expressed as a combination of dental tipping (orthodontic expansion) and sutural (skeletal) expansion along the midpalatal suture, currently no established methodology exists to assess the magnitude and breakdown of these forces. The aim of this study is to assess the feasibility of devising an appliance with sensors to facilitate the extraction of real-time information on forces in three dimensions expressed by activation of a hyrax RPE. A further aim of this work is to show that meaningful measurements are possible even in situations where the use of only one sensor is suitable or available. For example, some passive wireless sensors can operate on identical or closely spaced frequencies [18]. Such a situation would result in the readouts from individual sensors interfering with each other. Another example is one in which spatial constraints such as those in pediatric patients prevent the use of multiple sensors. Under these circumstances, it would be preferable to integrate a single sensor within the palatal expander. 

## 2. Materials and Methods 

### 2.1. Appliance Fabrication and Incorporation of Sensor 

**Figure 1:**
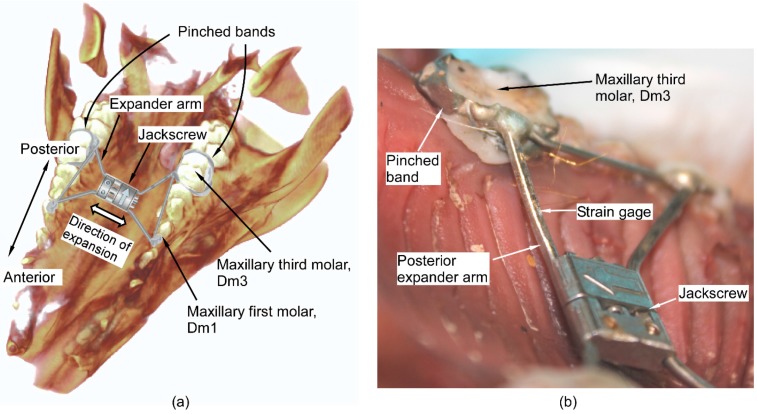
(**a**) Illustration of the hyrax-design RPE and the attachment of the expander arms superimposed on the maxillofacial structures of the pig head. (**b**) Location of the strain sensor attached to the posterior expander arm, prior to the use of dental composite.

A hyrax RPE (OrthoXPAND™ 7 mm jackscrew, Lakeville, IN, USA) was fabricated to fit the dentition and palate of the maxilla of a freshly euthanized 5-month old *Sus scrofa* (Swine Teaching and Research Center, Michigan State University, Lansing, MI, USA). Custom pinched bands were fitted on the maxillary third molars (Dm3) (Figure 1a). The bands were then soldered to the posterior arms of the RPE and to the corresponding anterior arms using a 316 stainless steel extension arm. The maxillary first molars (Dm1) molar on which the anterior arms would be typically anchored for a four-banded hyrax RPE were not banded due to inadequate eruption of these teeth. As a result, the anterior arms rested against the lingual surface of Dm1, but were not anchored to the teeth.

The posterior arm of the expander was affixed with a single axis, U-shaped p-doped silicon semiconductor strain gage (SS-018-011-3000PU, Micron Instruments, Simi Valley, CA, USA). To house the strain gage, a flat surface was machined on the arm using micro-electrodischarge machining (μEDM) [19]. The strain gage was attached using cyanoacrylate adhesive (Figure 1b) and then covered with light-cure Transbond XT (3M Unitek, Monrovia, CA, USA) dental composite. The custom hyrax-design expander was then cemented into place using light-cure OptiBand Ultra (Ormco Corporation, Orange County, CA, USA) dental composite. Leads were soldered to the sensors to measure resistance during expansion. The strain gage resistances were read in real-time using data acquisition device NI6341 and LabVIEW (National Instruments Corporation, Austin, TX, USA) at a sampling rate of 10 samples/s.

The deformation of the arms under applied activation of the jackscrew was measured using a strain gage, the data from which was used to estimate the expansion forces. The relationship between the arm deformation (and consequently, the strain on the arms) and the expansion forces was determined using finite element analysis (FEA). From this relationship and the measured strain data, the expansion forces on the arms could be estimated.

**Figure 2:**
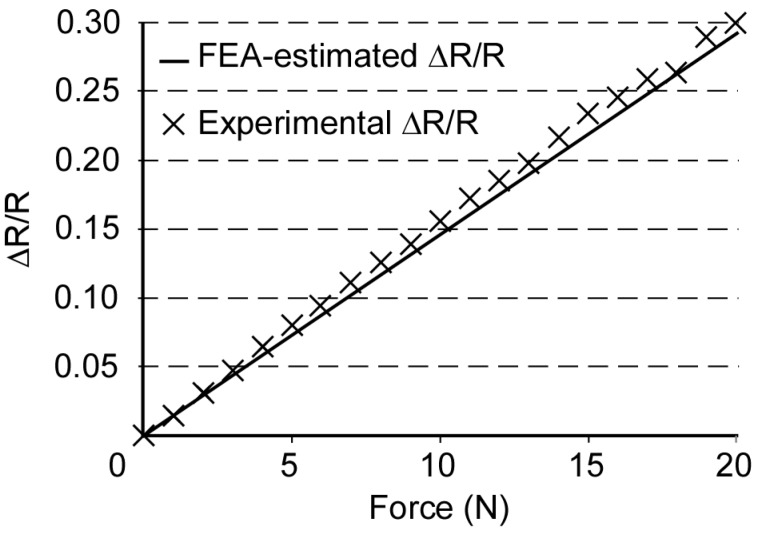
Comparison of experimental and FEA-estimated resistance change for an applied load on a test cantilever.

The validity of this approach was first confirmed on a simple cantilever instrumented with the strain gage. The cantilever was a metal bar with a 1.5 × 5 mm^2^ cross section and 30 mm length. The strain gage was attached to the cantilever which was then deflected with a prescribed load. The strains the cantilever was subject to were similar to those expected in a palatal expander. The change in resistance of the strain gage was recorded under this applied load. This measured change in resistance was compared to an FEA-estimated change in resistance. COMSOL Multiphysics software (COMSOL AB, Stockholm, Sweden) was used for the FEA to simulate the strains on a fixed cantilever under an applied load. In the FEA simulation, strain was averaged over the footprint of the strain gage to estimate the strain to which the strain gage was exposed. Consequently, the FEA-estimated strain closely approximates the strain as measured experimentally by the strain sensor. The corresponding FEA-estimated change in resistance of the strain gage was then calculated using the gage factor data provided by the manufacturer and the average FEA-estimated strain. Figure 2 shows the experimentally measured and FEA-estimated change in resistance values. The similarity between these validates the approach. In addition, this experiment also verifies the strain gage performance and a linear response is observed for up to 30% change in resistance.

### 2.2. Force Relationships

**Figure 3:**
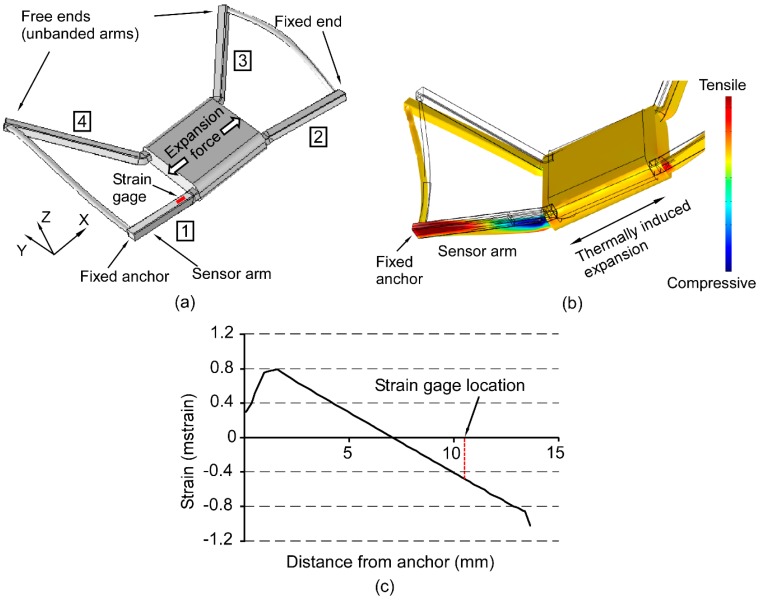
(**a**) 3D model of the expander used for FEA. The individual expander arms have been numbered. Arm 1 is instrumented with the strain gage. (**b**) FEA of strain along the expander arm for an arbitrary expansion. Compressive strain is observed at the location of the strain gage on the sensor arm (arm 1). (**c**) Strain on the top surface of the sensor arm as a function of distance from the fixed anchor.

In a palatal expander, the force applied to the expander arms can be resolved along three Cartesian coordinates. Building on the approach described in the previous section for the uniaxial case, the three-dimensional response is determined. The relationship between the strain on the posterior expander arm and the forces on the arms is determined using a 3D FEA. A 3D model of the expander was created in Dassault Solidworks (Dassault Systèmes, Vèlizy-Villacoublay, France) and imported into the COMSOL Multiphysics. As illustrated in Figure 3a, the expansion force acts outward axially from the central block of the expander where the jackscrew is located. In the case of the expander, the FEA simulation provides the strain resulting from the combination of bending, axial compression, torque, and shear and when averaged over the footprint of the sensor, as in the simulation of the test cantilever, it is representative of the measured strain.

The dimensions and material properties of the expander are listed in Table 1 and Table 2. The mechanical displacement effected by the jackscrew during the expansion was emulated by a thermally induced expansion. In this method, which follows commonly accepted practice, an artificial temperature rise combined with an assumed expansion coefficient in the constituent material are used to mimic strain resulting from other forms of actuation that cannot be replicated in the model. The expansion caused by the jackscrew was mimicked by defining a temperature rise at the jackscrew in combination with a predefined expansion coefficient of the jackscrew. The boundary conditions on the expander arms replicated the test conditions in the pig head; the posterior arms were attached to fixed supports, while the unbanded anterior arms were assumed to be unconstrained.

In the simulation model, the strain gage was attached 11 mm away from the fixed end (which is anchored to the tooth) on the sensor arm (Figure 3a). The strain along the axis of the sensor arm, *i.e.*, arm 1, instrumented with the strain gage, for an arbitrary expansion is plotted in Figure 3b. Figure 3c shows the variation of strain along the surface of the expander arm as a function of the distance from the fixed anchor for an arbitrary expansion. The strain gage was attached at a location that undergoes compressive strain upon jackscrew activation. The strain gage was attached in the region where the strain was high, on the part of the expander arm which was straight and away from the bend (where it was attached to the jackscrew). The region of the expander arm (near the center) where the strain is estimated to be zero is avoided. 

A fixed boundary condition was assumed at the anchors to the teeth [13]. This is acceptable because maximum compliance of the palatal expander is almost exclusively along the direction of expansion. The hyrax screw provides expansion along one axis, and the expander is relatively stiff along the other axes. Hence, the strain measurement is less sensitive to modest variations in boundary conditions and a fixed boundary condition can provide a good approximation in finite element studies.

Based on this simulation, the empirical relations between the average magnitude of strain at the location of the sensor and the expansion forces are approximately:

(1) $$F_{total}=4.7371\times 10^{4}\times e_{gage}$$

(2) $$F_{x}=4.7236\times 10^{4}\times e_{gage}$$

(3) $$F_{y}=1.350\times 10^{2}\times e_{gage}$$

(4) $$F_{z}=3.573\times 10^{3}\times e_{gage}$$

where, *F_total_*, *F_x_*, *F_y_*, and *F_z_* are the total force and forces along the *x*, *y* and *z* axes respectively, whereas *e_gage_* is the average magnitude of strain at the location of the sensor. 

Although the simulation assumed the arms to be anchored to fixed constraints, in reality, the dental tipping and skeletal expansion results in movement of these anchors as well. Hence, the expansion assumed in simulation would, in actuality, correspond to the difference between the jackscrew activation and the combined result of dental tipping and skeletal expansion.

The expander was subjected to a total of 8 quarter-turns of activation of the jackscrew over a duration of 1900 s (totaling 2.0 mm), separated by 200–300 s intervals. The activation of the RPE’s jackscrew depends on the number of turns applied to it. The compression of the arms (*d_arms_*) is the difference between the RPE’s jackscrew activation (*d_act_*) and resulting expansion (*d_exp_*), and is reflected in the strain gage reading. The FEA yields an empirical formula for *d_arms_*:

(5) $$d_{arms}\left(\mathrm{in}\mathrm{mm}\right)=165\times e_{gage}$$

The resulting expansion for a given jackscrew activation can now be expressed as:

(6) $$d_{exp}=d_{act}-d_{arms}$$

While it may be postulated that variations in anatomy will lead to differing force distributions, these variations are accommodated by customized construction of the expander to each patient’s palatal and dental anatomy. Further, the empirical relations defined in Equations (1)–(4) hold true only for the specific boundary conditions of the case study described in this work. Under different conditions, the FEA results may represent different boundary conditions, and the empirical equations would be modified accordingly. 

### 2.3. Outcome Evaluation

Three-dimensional imaging with cone beam computed tomography (CBCT) was used to quantify the post-expansion changes in the midpalatal suture and rebound in the dentition. CBCT was performed with 3D Accuitomo 170 (J Morita USA, Irvine, CA, USA) on the fresh pig maxillofacial structures just after the expander was removed and again after three days of relapse. The CBCT images were captured using the following settings: 17 × 12 cm^2^ field of view, high-fidelity (360°) scan mode, 90 kV and 5 mA for 30.8 s with a voxel size of 0.25 mm. 

## 3. Results

**Figure 4:**
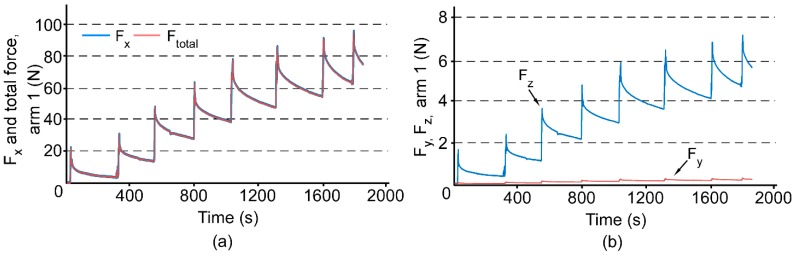
(**a**) Total force (*F_total_*) and force along *x* axis (*F_x_*), and (**b**) *F_y_* and *F_z_* on the sensor arm (arm 1) for a total of eight turns of the jackscrew. *F_x_* is dominant component of the total force and *F_y_* is negligibly small.

The expander was subjected to a total of 2 mm of activation, over a duration of 1900 s. Using LabVIEW, the strain measurements were recorded for the entire duration of the experiment. The strain measurements were translated to expansion forces using Equations (1)–(4) and plotted as a function of time (Figure 4).

Figure 4a shows the total force (*F_total_*) and *F_x_*, which is the expansion force in the transverse dimension across the midpalatal suture. This figure confirms that *F_x_* is the overwhelming contributor to the total expansion force, and the two are indistinguishable in the plot. In each quarter-turn, *F_x_* first spikes by an average of 34 N and then reduces by an average of 24 N in the 200–300 s interval between turns. At the end of the activation sequence, *F_x_* builds up to a peak of 98 N. In contrast to *F_x_*, *F_y_* and *F_z_* are the expansion forces in the anterior-posterior dimension and the vertical dimension, respectively. These forces are much smaller than *F_x_*, with *F_z_* peaking at 7 N (Figure 4b). Force *F_x_* is negligibly small because the anterior arms of the expander are not anchored.

**Figure 5:**
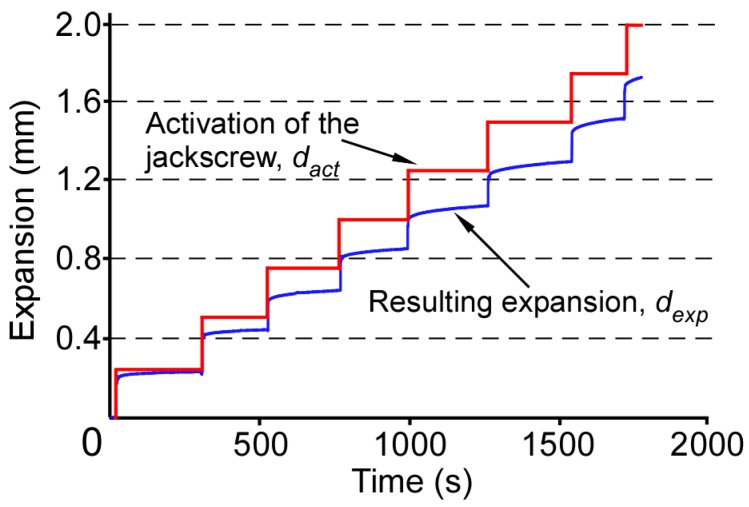
Comparison of the activation of the jackscrew (*d_act_*) and resulting expansion (*d_exp_*) as a function of time for eight turns of the jackscrew (2.0 mm total activation).

The resulting expansion, *d_exp_*, is determined using the strain gage readings and Equations (5) and (6) (Figure 5). Almost 85% of the activation is immediately transferred to the palate and teeth.

**Figure 6:**
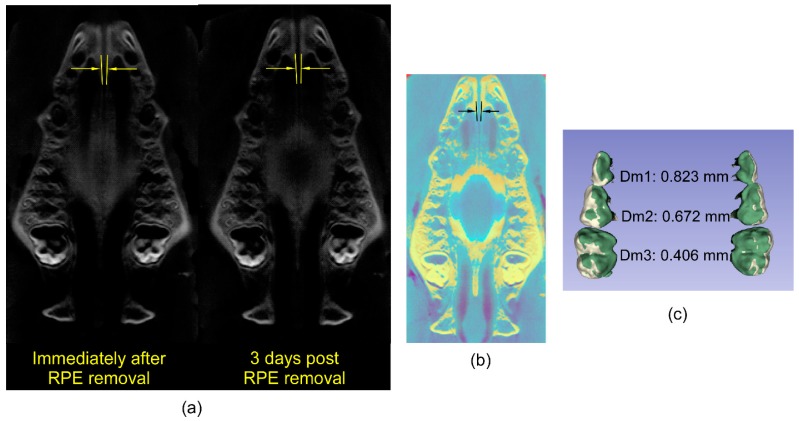
(**a**) Axial slices of CBCT images obtained immediately after RPE removal (left) and after three days of relapse (right) showing sutural separation demarcated by lines and yellow arrows). (**b**) Generalized superimposition of the palate immediately after RPE removal (cyan) and after three days of relapse (yellow) showing minimal changes in midpalatal suture separation. (**c**) Generalized superimposition of the maxillary dentition immediately after RPE removal (white) and after three days of relapse (green) showing the magnitude of relapse at the first (Dm1); second (Dm2) and third (Dm3) molars.

A comparison of the suture at the level of the palate immediately after removal of the RPE and three days post-removal is presented in Figure 6a. These two axial slices from the different time points are superimposed in Figure 6b using a regional superimposition of the maxilla. As evident from the lack of suture closure in the three day time period, there was little skeletal relapse. This is consistent with the non-responsive nature of the fresh carcass. Although the skeletal relapse is negligible, the dental relapse is evident. As shown in Figure 6c, this varies from 0.4 mm at the third molar to 0.82 mm at the first. 

## 4. Discussion

The acute activation of the expander accounts for the high force (almost 100 N) observed in the experiments. Generally, 24 h is prescribed between activations of one quarter-turn or 0.25 mm of expansion of the expander. This is in contrast to the 200–300 s delays in our experiment. The total force of this experiment cannot be directly compared with that of Isaacson *et al.* [14] because of the differing species and experimental methods. However, the rapid rise in force levels with a non-linear decay pattern is consistent with previous findings. Since this was a proof of concept study, the interval between activations and the high magnitude of forces of the experimental time frame was considered less critical to the analyses than determining the expression of forces in three dimensions.

As evident from Figure 5, a portion of the given activation of the jackscrew results in immediate expansion which is then followed by a gradual increase in the expansion. Consequently, the difference between the applied activation and resulting expansion progressively decreases in the time intervals following an activation. An interval of sufficient duration will permit the expansion to plateau before the next activation.

The dental relapse observed in Figure 6c shows that dental tipping is a component of total expansion, in addition to the skeletal expansion, confirming previous observations [2,7,9]. This is expected as the RPE used is a tooth-borne device. Taken together, the sutural patency and dental rebound demonstrate that resistance to expansion offered by skeletal and periodontal tissues contribute to the total expansion force.

Whereas semiconductor strain gages provide excellent sensitivity, such strain gages exhibit high thermal coefficients of resistance and of sensitivity. However, because the experiments reported in this work were performed at a constant room temperature of 21 °C, the thermal effects on the results are expected to be minimal. For *in vivo* applications, the temperature is expected to remain close to body temperature and substantial variations of temperature are not expected. For instance, a 1 °C change in temperature would result in a 11 Ω change in resistance, which is equivalent to an approximate strain of 25 μstrain for the SS-018-011-3000PU used in this study. This corresponds to merely 1.18 N of total force, *F_total_*. 

Based on the simulations, it is estimated that a positional error of 0.5 mm in the sensor location, which was measured at 11 mm away from the fixed end, would result in a variation of 15% in the measured strain reading. For the purpose of this work, this is an acceptable margin of error, particularly given that only one strain sensor is necessary. Use of multiple sensors could improve the measurement accuracy at a cost of possible increased interference between wireless sensors or increased size of the overall setup. 

From a clinical perspective, these results represent a significant step toward detailing the force distributions of a conventional tooth-borne RPE. From an engineering perspective, the results demonstrate a means of measuring the transactivation force of a RPE *in situ* and the resulting expansion. Taken together, these findings provide the basis for further development of smart orthodontic appliances that provide real-time readouts of forces and movements, which will allow personalized, optimal treatment for the first time.

## 5. Conclusions 

This work describes the first real-time quantitative measurements of three-dimensional forces associated with RPE in all three planes of space. This is accomplished using a single sensor, which minimizes the challenge of sensor integration and electronic read-out. The simulation itself can be expanded to include a complete 3D model of the skull and variations in the type of expander used to predict the treatment outcomes for a given expansion protocol. Real-time force measurement during the treatment can then be used to track the patient response and modify the protocol, if needed. In a broader sense, this work develops the methodology and establishes a proof of concept towards achieving the basic foundations needed for our longer-term goal of developing customized expanders whose activation can be individualized both in magnitude and direction for each patient.

A complete force measurement system for this application would include a wireless readout circuit. Fully integrated wireless sensing systems with packaging for compatibility and sterilization have been previously developed for other applications [20]. Biocompatibility of the transducer can be enhanced by coating it with Parylene—a biocompatible polymer [21].

Commercially available expanders also include bone-anchored RPEs, which eliminate the dental movement component of expansion seen with tooth-borne RPEs. Future studies will include determination of forces and movements generated with these devices without the complexities of dental movements. Future research will also include *in vivo* testing incorporating CBCT imaging before, immediately after and post-expansion with a longer decay interval between expander activations to better simulate human biological responses to expansion forces.

## Figures and Tables

**Table 1:** Dimensions of the expander arms.

Arm Number	Angle with *xy* Plane	Angle with *xz* Plane	Length (mm)
1	29°	21°	13.8
2	21°	29°	12.3
3	24°	33°	16.5
4	25°	43°	16.9
Cross section (all arms)		1.2 × 1.2 mm^2^	

**Table 2:** Material properties (316 stainless steel).

Young’s Modulus	Poisson’s Ratio	Density
193 GPa	0.27	8000 kg/m^3^
